# Spatial Transferability of Expansion Factors for Estimating Pedestrian Volume at Intersections

**DOI:** 10.1177/03611981251350639

**Published:** 2025-09-08

**Authors:** Lucas Tito Pereira Sobreira, Bruce Hellinga

**Affiliations:** 1 Department of Civil and Environmental Engineering, University of Waterloo, Waterloo, ON, Canada

**Keywords:** Pedestrian volume, Expansion factors, Spatial transferability, Factor groups

## Abstract

Pedestrian exposure at intersections is a key input for jurisdictions to develop pedestrian-centric strategies. Exposure is typically expressed as the annual average daily pedestrian traffic (AADPT). The expansion factor method is the most common technique employed to estimate AADPT, consisting of the expansion of short-term counts (STCs) using factors calculated from sites with continuous counts (CCs). However, jurisdictions often lack sufficient CCs to determine expansion factors, which motivated this work of assessing the spatial transferability of expansion factors. This paper examines the spatial transferability across three jurisdictions in Ontario, Canada, with similar school holiday and weather seasonality, and one jurisdiction in Arizona, U.S.A., with different characteristics. It was assumed that the “base jurisdiction” has CCs available, while the “target jurisdiction” has only STC sites, and applies expansion factors from the base jurisdiction. Two approaches were tested: the single factor method, averaging expansion factors across all sites in the base jurisdiction, and modeling methods, which assign STC sites to factor groups based on models developed in the base jurisdiction. The single factor method showed acceptable transferability within the Ontario jurisdictions, with an average absolute increase of 2.3% (8.7% relative increase) in the mean absolute percent error of AADPT achieved when comparing the spatially transferred factors to those obtained when the base and target jurisdictions are the same. However, poor transferability was observed between jurisdictions with differing characteristics. The application of modeling methods yielded inconsistent results, possibly because of the limited number of sites available in this study, and requires further investigation.

Improving safety for vulnerable road users, such as pedestrians and cyclists, is crucial to the successful implementation of Vision Zero programs (*1*). An important step in mitigating or eliminating fatal and injury road crashes is the deployment of the roadway safety management process proposed in the Highway Safety Manual (*2*), which guides jurisdictions in prioritizing sites for the implementation of countermeasures. This management process relies on safety assessments of the entire road network, which, with respect to pedestrian safety, depend on the availability of pedestrian exposure data (*3* [Bibr bibr4-03611981251350639]–*5*). Given this importance, the scope of this work is the estimation of pedestrian exposure (or volume) at intersections.

Two typical sources of pedestrian volume data at intersections available for jurisdictions are short-term counts (STCs) and continuous counts (CCs). STCs are often collected for traffic signal timing purposes (often termed turning movement counts) and represent vehicular, cyclist, and pedestrian counts over a given number of hours on one or a few weekdays. Unlike STCs, CCs offer long-term monitoring of pedestrian activity at intersections. This allows for the identification of hourly, weekly, monthly, and seasonal patterns, which is crucial for employing the expansion factor method to adjust STCs to estimate long-term volumes, such as the annual average daily pedestrian traffic (AADPT).

The existence of different pedestrian volume patterns across a jurisdiction (*6* [Bibr bibr7-03611981251350639]–*8*) necessitates identifying sites with similar patterns, referred to as factor groups, to ensure the proper application of the expansion factor method. This approach enables more precise application of expansion factors, rather than simply averaging them across all sites with available CC stations. Identifying such patterns in sites with CC stations is straightforward because of continuous monitoring. The challenge arises when associating STC sites—where temporal patterns are unknown—with factor groups. A common solution is to develop models linking land use, socioeconomic, and transportation attributes with factor groups to address this challenge (*9* [Bibr bibr10-03611981251350639]–*11*).

Fundamentally, the application of the expansion factor method requires jurisdictions to have several sites with continuous monitoring. Grossman et al. (*12*) surveyed 133 U.S. state, regional, and local transportation agencies and found that 84.2% of them do not have any CC stations that monitor pedestrian activity. This means that these jurisdictions are not able to develop expansion factors without implementing CC stations. In this context, one potential solution would be to apply expansion factors developed in another jurisdiction. To our knowledge, there is a gap in the literature with respect to the spatial transferability of expansion factors for pedestrians (and other types of road users as well).

Aside from spatially transferring expansion factors from another jurisdiction, there are alternative methods for estimating pedestrian exposure when no continuous monitoring exists. These methods include the following: (a) hiring third-party companies that use crowd-sourced data to estimate pedestrian volumes (*13*); (b) applying large language models to rank sites based on pedestrian activity and combine the rankings with traffic counts to estimate pedestrian volumes across the entire ranking (*14*); and (c) spatially transferring direct-demand (DD) models developed in another jurisdiction to estimate pedestrian volume as a function of land use, socioeconomic, and transportation attributes (*15*, *16*).

Considering (a) the frequency of jurisdictions without CC stations in the U.S.A. (and likely worldwide) and (b) the literature gap on this topic, the objective of this work is to investigate the spatial transferability of expansion factors for estimating AADPT at intersections. More specifically, this work aims to answer the following practical questions a practitioner would face when attempting to estimate pedestrian exposure from STCs and without the ability to compute expansion factors for the study jurisdiction.

1. Does the application of expansion factors spatially transferred from another jurisdiction provide sufficiently accurate AADPT estimates to be of practical value?
2. Does transferring models for associating STC sites with factor groups provide improved AADPT estimation accuracy that is of practical benefit as compared to using a single average factor group when transferring expansion factors?
3. How does the accuracy of spatially transferring expansion factors compare with that of alternative methods (e.g., crowd-sourced data, large language models, and DD models) when continuous monitoring is not available?

The next section presents a literature review on the expansion factor method and on alternative methods to estimate pedestrian exposure at intersections.

## Literature Review

### Expansion Factor Method

The application of the expansion factor method for pedestrian traffic volume has been widely documented in the literature using various strategies. Different aggregations for expansion factors have been considered, such as hour-to-week (*9*, *10*), day-of-week and month-of-year (*8*, *17*), day-of-week-of-month (DOWOM) (*6*, *7*, *11*), and day-of-year (*8*). Hankey et al. (*8*) demonstrated superior performance when using day-of-year factors, particularly because they capture daily variations in weather and other attributes. However, practical application of day-of-year factors poses challenges, including spatial transferability to other jurisdictions and the requirement for STCs to be available in the same years as CCs. Another topic explored is the effect of the duration of STCs on expansion performance (*6*, *8*, *9*). As expected, longer STCs (e.g., 12-h versus 2-h or 7-day versus 1-day) reduce errors when performing the expansion. The expansion factor method yields mean absolute percent errors (MAPEs) in the range of 15–30% (*6*, *8*, *11*).

Different pedestrian volume patterns are observed within a jurisdiction (*9* [Bibr bibr10-03611981251350639]–*11*). For example, sites near schools typically have reduced pedestrian volume during school holidays and weekends, while sites near industrial areas may experience reductions on weekends. The existing patterns within a jurisdiction can be identified from sites with continuous monitoring, enabling the classification of sites into factor groups with similar traffic patterns. In an ideal application of the expansion factor method, factors are calculated for each factor group and then the approach factor group expansion factor is applied to the STC from each STC site.

One challenge lies in associating STC sites with a given factor group, because the temporal traffic patterns at these sites cannot be observed from the short duration STCs. A common solution is to employ traffic indicators extracted from the STCs, such as those proposed by Miranda-Moreno et al. (*18*): WWI (relative index of weekend and weekday volume) and AMI (relative index of AM peak and mid-day period volume). Johnstone et al. (*6*), Nordback et al. (*7*), and Hankey et al. (*8*) are examples of studies that used only AMI, only WWI, and both AMI and WWI, respectively, for identifying factor groups of STC sites. However, there are a couple of limitations related to this strategy: (a) the application of the WWI requires STCs from both weekdays and weekends, which may not be available for all jurisdictions, and (b) these traffic indicators do not account for seasonality effects, which are an important attribute when characterizing pedestrian traffic patterns (*11*).

Modeling approaches have been implemented to address the limitations of using traffic indicators to link STC sites to factor groups. Multinomial logistic regressions (MLRs) have been employed to associate factor groups with land use, socioeconomic, and transportation attributes, allowing the association of STC sites to a given factor group without the need for knowledge of their temporal pedestrian traffic patterns. This approach was applied to hour-to-week (*9*, *10*), month-of-year (*10*), and DOWOM (*11*) expansion factors. For example, Medury et al. (*9*) linked four factor groups, based on hour-to-week expansion factors, to various attributes such as the presence of schools, park area, percentage of office buildings, and percentage of entertainment venues, among others.

Although the classification accuracy of factor groups employing the MLR models was around 70%, Sobreira and Hellinga (*11*) and Medury et al. (*9*) observed modest relative improvements in the MAPE of estimated AADPT (7% and 14%, respectively) when using the MLR method compared to a single averaged expansion factor. One may argue that these improvements are not of practical value, considering that the MLR method requires a substantial number of CC sites for calibration (i.e., approximately 10 sites per factor group) along with the expertise and geographic information system (GIS) information for model development.

The present study explores the spatial transferability of expansion factors for jurisdictions without readily available CC stations (Practical Question 1). It also evaluates whether transferring models to associate STC sites with factor groups offers practical benefits compared to using a single averaged factor group for transferring expansion factors (Practical Question 2).

### Alternative Methods to Estimate Pedestrian Exposure

In addition to spatially transferring expansion factors from another jurisdiction, alternative methods exist for estimating pedestrian exposure in the absence of continuous monitoring. However, these methods—although often less resource-intensive—yield larger errors compared to those observed with the expansion factor method. Some of these methods are described below.

One method involves hiring third-party companies that provide volume estimates based on crowd-sourced data. For example, the company StreetLight uses anonymized location data collected from various mobile apps to estimate pedestrian volume (*13*). From a jurisdictional perspective, using this type of data is convenient as it eliminates the need for direct data collection, although it may come with significant costs. With respect to performance, the method generally achieves MAPEs ranging between 71% and 151%, depending on the magnitude of pedestrian volume of the studied sites.

Another method employs large language models, such as ChatGPT Vision 4, to rank sites based on pedestrian activity derived from satellite imagery (*14*). The generated ranking is then combined with traffic counts at specific sites to estimate pedestrian volume metrics (e.g., daily volume, weekly volume, or AADPT) for all sites in the ranking. This approach is relatively resource-efficient, requiring no extensive data collection or reliance on land use and socioeconomic GIS databases. The method yields MAPEs ranging from 49% to 273%, depending on the magnitude of pedestrian volume.

A third method involves the spatial transferability of DD models developed in another jurisdiction. DD models predict pedestrian volume based on attributes of the intersection's surroundings, such as land use, socioeconomic factors, and transportation characteristics. Sobreira and Hellinga (*15*) tested the transferability of DD models developed in the U.S.A. to different jurisdictions, observing varying performance. The mean absolute error (MAE) normalized by average observed AADPT ranged from 0.52 in cases of acceptable transferability to values greater than 3.00 in cases of poor transferability.

An extension of this method involves leveraging pedestrian volume data available within the study jurisdiction to enhance the transferability of an existing DD model from another jurisdiction (*16*). This process, known as local calibration, has shown promise in improving DD model transferability when insufficient local data is available to develop a new model. Using this approach, a normalized MAE of approximately 0.65 was achieved.

This study aims to compare the performance of the expansion factor method with spatially transferred factors against the aforementioned alternative methods (Practical Question 3). The next section presents the jurisdictions considered in this study.

## Study Sites

This work was developed considering four jurisdictions. Three of them are located in Ontario, Canada: the Region of Waterloo (including the cities of Cambridge, Kitchener, and Waterloo), the City of Toronto, and the City of Milton. These jurisdictions are within a similar range of latitude and share comparable weather conditions: four distinct seasons, including cold winters (average daytime temperatures ≤0°C [32°F] from December to February) and hot summers (average daytime temperatures ≥24°C [75°F] from June to August) (*19*). In addition, these jurisdictions have similar school holiday periods in July and August. The fourth jurisdiction is Pima County, Arizona, U.S.A., which experiences different weather conditions compared to the Ontario jurisdictions (i.e., milder winters and hotter summers) and has distinct school holiday periods in June and July.

Given the differences among the jurisdictions, it is expected that expansion factors spatially transferred across the jurisdictions in Ontario will perform better than those from Pima County. We acknowledge that, from a practical perspective, one would not typically consider transferring expansion factors from Pima County to Toronto, for example. However, we included Pima County to represent a scenario where poor transferability is likely to be observed.

Table 1 presents the number of sites and descriptive statistics of the AADPT in each jurisdiction. The average AADPT highlights the differences in pedestrian activity between jurisdictions. Pima County and Toronto exhibit very low and very high pedestrian activity, respectively. This variation is explained by the site locations used in this work: none of the sites from Pima County are in the City of Tucson (the most populated city in the county), while 19 out of the 34 sites in Toronto are in the Downtown area, which has very high pedestrian activity. Milton and Waterloo present mid-levels of AADPT.

**Table 1.** Descriptive Statistics of Annual Average Daily Pedestrian Traffic (AADPT)

		AADPT			
Jurisdiction	#Sites	Average	Standard deviation	Minimum	Maximum
Waterloo	101	852	1152	16	6146
Toronto	34	14,282	10,557	1283	33,629
Milton	33	350	265	26	1124
Pima county	20	52	50	16	202

Expansion factors are calculated as the ratio between AADPT and short-term volumes (further discussed in the next section). Therefore, differences in overall pedestrian activity levels across jurisdictions do not necessarily imply that expansion factors are unsuitable for transferability. If jurisdictions exhibit similar temporal patterns, transferred expansion factors may still yield reasonable accuracy, even if pedestrian activity levels differ significantly. This is why Ontario jurisdictions may have transferable expansion factors: as they share similar weather and school holidays, they may also share similar temporal patterns.

This work considered sites equipped with camera-based CC stations, all located at signalized intersections, with the analysis period spanning from April 1, 2023, to March 31, 2024. Filters were applied to ensure that CC stations were collecting data properly and to remove outliers.

1. Days of proper functioning: at least 72 out of 96 15-min count intervals had to register at least one motorized vehicle, and the total daily pedestrian volume had to be greater than 0.
2. Outlier removal: for each month and each site, a filter was applied to the pedestrian counts to remove outliers. This involved excluding daily (24-h) pedestrian volumes that were either (a) above the third quartile plus 1.5 times the interquartile range, or (b) below the first quartile minus 1.5 times the interquartile range.

After applying the filters, the average number of days available for each site was 359, with a minimum of 242 days. Of the 188 sites, only 14 had fewer than 350 days of data available.

## Framework

Before presenting the framework, two key terms are defined: base jurisdiction and target jurisdiction. The base jurisdiction refers to the jurisdiction where CCs are available. Expansion factors are calculated in this jurisdiction and then transferred to the target jurisdiction. In the target jurisdiction, it is assumed that only STC sites are available.

**Figure 1.**
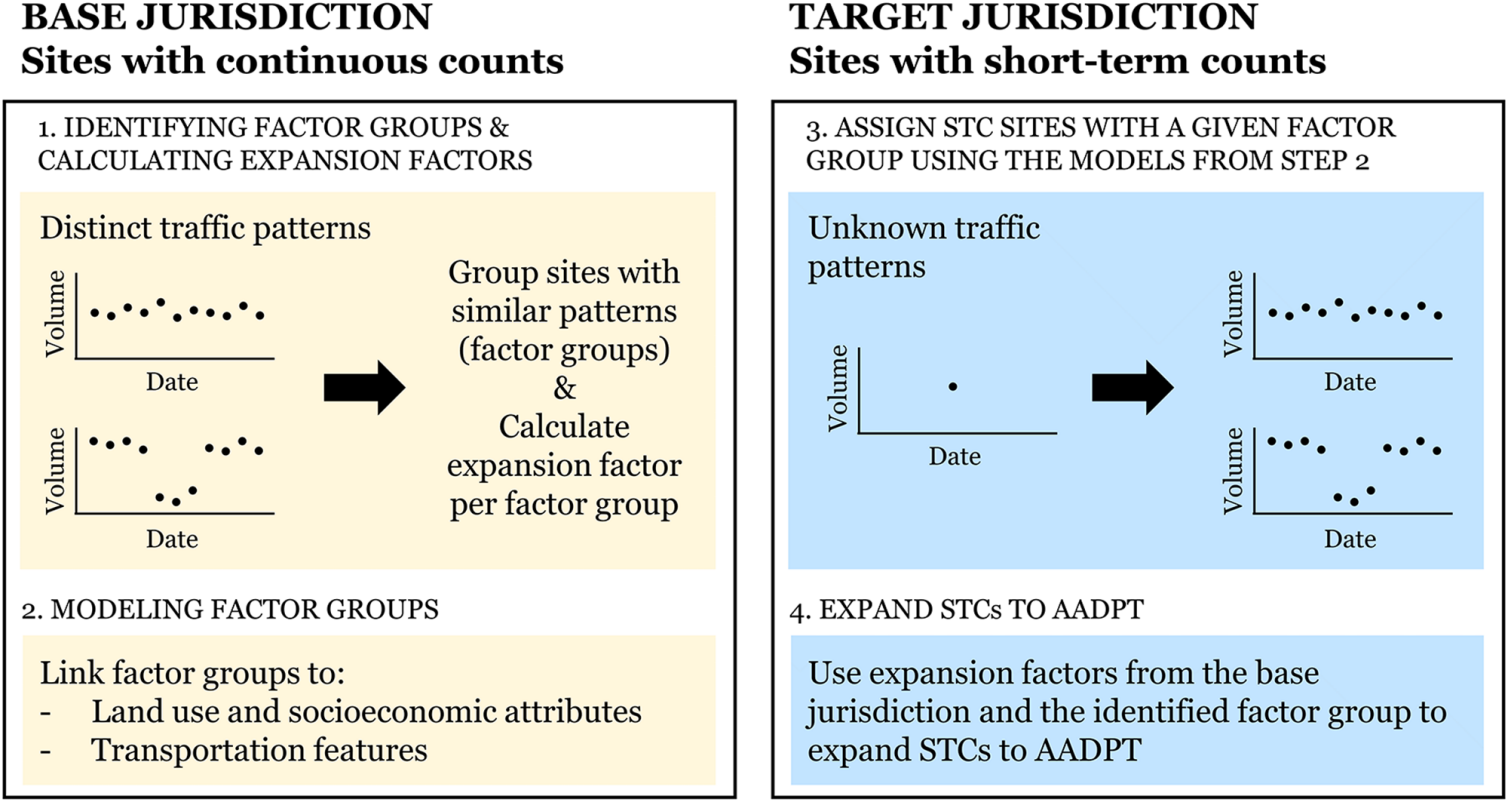
Framework. *Note*: AADPT = annual average daily pedestrian traffic; STC = short-term count.

Figure 1 displays the proposed framework for spatially transferring expansion factors. The framework involves four major steps: two in the base jurisdiction and two in the target jurisdiction. The first step is the identification of factor groups and the calculation of expansion factors in the base jurisdiction. The second step consists of modeling factor groups with the goal of allowing the association of STC sites with a given factor group. Moving to the target jurisdiction, the third step involves assigning STC sites with a given factor group using the models developed in the second step. In the final step, the STCs in the target jurisdiction are expanded to AADPT using the expansion factors retrieved from the base jurisdiction and the associated factor group.

Note that Steps 1–3 are strictly related to Practical Question 2, which examines whether transferring models to associate STCs with factor groups provides practical benefits. The application of single expansion factors averaged across all sites (i.e., one unique factor group) does not require these steps. To emphasize the discussion of the three practical questions while reducing the complexity and density of this document, we have presented the methods and results for Steps 1–3 in the appendix while including an overview in the main body. Step 4 represents the most critical portion of this study and is linked to all three practical questions; therefore, its methods and results are presented in the body of the document.

The remainder of this document is organized as follows: the next section introduces the types of expansion factors employed, followed by an overview of Steps 1–3. Subsequently, the methods and results for Step 4 are presented. Finally, the document concludes with a discussion of the three practical questions, along with conclusions and practical recommendations.

## Day-of-Week-of-Month Expansion Factors

This work employs DOWOM expansion factors to expand 8-h STCs collected during the periods 7–9 a.m., 11 a.m.–2 p.m., and 3–6 p.m. The method for calculating these expansion factors involves first computing the AADPT for each site (Equation 1). Next, the expansion factor for each of the 84 DOWOM factors is calculated for each site (Equation 2). When factor groups are used, the expansion factors for each group are determined as the average of $EF_{i,m,d}$ for sites within that group. In cases where a single factor group approach is applied, the average of $EF_{i,m,d}$ across all sites is used. Note that Equation 1 can only be applied for site *i* when there is at least one (i.e., $n_{i,m,d}\geq 1,\forall \;m,d$ ) observed daily pedestrian volume count for each day of week (*d*) in each month of the year (*m*). All sites used in this study satisfied this condition:

(1) $$\mathrm{AADP}T_{i}=\frac{1}{12}\sum_{m=1}^{12}\left[\frac{1}{7}\sum_{d=1}^{7}\left(\frac{1}{n_{i,m,d}}\sum_{k=1}^{n_{i,m,d}}V_{i,k,m,d}^{24}\right)\right]$$

(2) $$EF_{i,m,d}=\frac{\mathrm{AADP}T_{i}}{\frac{1}{n_{i,m,d}}\sum_{k=1}^{n_{i,m,d}}V_{i,k,m,d}^{x}}$$

where $\mathrm{AADP}T_{i}$ is the AADPT of intersection i,m is the month (1–12), d . is the day of the week (1–7), $n_{i\;,\;m,\;d}$ . is the number of times a specific day of the week occurs in a specified month, and $V_{i,k,m,d}^{24}$ is the daily (24-h) pedestrian volume of site i on the *k*th occurrence of month ( m ) and day of the week ( d ). For example, if there are three observations on Mondays in January, then *k* will range from 1 to 3. Further, $EF_{i,\;m,\;d}$ . is the expansion factor of site i for month ( m ) and day of the week ( d ), and $V_{i,k,m,d}^{x}$ is the pedestrian volume of site i on the *k*th occurrence of month ( m ) and day of the week ( d ) for duration x ( x = 8 h in this case).

## Steps 1–3: Overview

Step 1 involves identifying factor groups at CC sites within the base jurisdiction. Three traffic indices were used to characterize the temporal patterns at each site, followed by a clustering technique to group sites with similar characteristics. Figure A1 illustrates the factor groups for each jurisdiction.

Similar factor groups were identified in Waterloo and Toronto: (a) factor group #1: multipurpose sites with relatively constant pedestrian activity throughout the year; (b) factor group #2: commuter sites with reduced volumes on weekends; and (c) factor group #3: sites primarily affected by schools, showing reduced activity on weekends and during school holiday months. Milton exhibited only factor groups #1 and #3. Pima County resulted in three factor groups, but with characteristics different from those in the Ontario jurisdictions.

Step 2 involves associating the factor groups identified in Step 1 with land use, socioeconomic, and transportation attributes. Because of small sample sizes and differences in pedestrian activity patterns compared to Ontario, this step was not applied to Pima County.

For the Ontario sites, four different models were considered. The first used MLRs for cases with more than two factor groups. The remaining models focused on distinguishing factor group #3 (associated with schools) from the others. These included binary logistic regression (LR) and simple threshold-based models, which identified sites in factor group #3 based on the presence of schools and the combined presence of schools and non-dominant commercial land use. Because of a limited sample size, MLR and LR models were not developed for Toronto. Tables A1 and A2 summarize the models developed for Step 2.

Step 3 switches to the target jurisdiction, where only STC sites are available. The models developed in Step 2 for the base jurisdiction are transferred and applied to the target jurisdiction to assign each site to a specific factor group. Transferability performance was inconsistent, with some models transferring well to certain jurisdictions and others performing poorly. Table A3 summarizes the transferability results.

Again, detailed explanations of the methods and results from Steps 1–3 are provided in the appendix. The next section focuses on Step 4, which represents the actual application of the expansion factor method in the target jurisdiction.

## Step 4: Expanding Short-Term Counts to Annual Average Daily Pedestrian Traffic in the Target Jurisdiction

### Method

Five different methods were employed to expand STCs. The first method expands the 8-h counts to daily volumes, while the others expand to AADPT. The first three methods are only applicable when the base and target jurisdictions are the same; these methods serve as a reference for comparing the spatial transferability considered in the remaining methods. The methods are described below.

1. Ratio 24/8 method: this method applies when no CCs are available in the target jurisdiction, and no expansion factors can be transferred from another jurisdiction. To expand 8-h STCs to daily (24-h) volumes in this scenario, a simple solution is to collect 1-day 24-h volume data at a selected number of sites (*k*). This allows for the calculation of an average "ratio 24/8" factor, which is then applied to STCs. We tested using *k* = 5 and *k* = 10 sites and found that both yielded similar results; therefore, we adopted *k* = 5 for the remainder of this paper. Because of the inherent variability in selecting the five sites and the days for calculating the ratio, the procedure was repeated 100 times, with the results presented as an average. Theoretically, this method represents the "worst-case scenario" for estimating daily pedestrian exposure.
2. From STC site method: in this method, both the STC and the expansion factor come from the same site, meaning that each site represents one factor group. This method cannot be used in practice because expansion factors can only be computed from CC sites and not from STC sites. It represents the "best-case scenario.”
3. Empirical factor group method: in this method, the observed (or true) factor group for each site, as obtained in Step 1, is used. Although this method cannot be applied in practice (since clustering based on temporal indicators is only possible for CC sites, not STC sites), it eliminates modeling error. This allows for the identification of sources of error, such as issues with combining sites into factor groups and assigning STC sites to factor groups via models.
4. Single factor method: this method uses a single expansion factor for each DOWOM factor, determined by the average of expansion factors across all sites (i.e., one unique factor group).
5. Modeling methods: for the school-based models, a specific factor group is predicted for each site. In the case of MLR and LR models, probabilities of a site belonging to a specific group are available. Therefore, two strategies can be used: (a) maximum probability: select the expansion factor from the factor group with the highest probability, and (b) weighted probability: use an expansion factor calculated as the weighted average of the DOWOM expansion factors from each factor group, with weights derived from the probabilities provided by the MLR and LR model outcomes. Medury et al. (*9*) and Sobreira and Hellinga (*11*) found that the weighted probability strategy performed slightly better; thus, it was adopted in this work.

This work uses sites with CCs because of the necessity of knowing the observed AADPT at each site to evaluate the quality of the STC expansion. Multiple STCs can be retrieved from each site, resulting in multiple predicted AADPT estimates (Equation 3). The performance of each individual prediction is assessed using the absolute percent error ( $\mathrm{AP}E_{i,j}$ —Equation 4) and then averaged across all observations j and sites i to obtain the MAPE (Equation 5):

(3) $${\mathrm{AADPT}}_{i,j}^{\prime }=EF_{i,m,d}\;\times \;V_{i,j,m,d}^{x}$$

(4) $$\mathrm{AP}E_{i,j}=\left|\frac{{\mathrm{AADPT}}_{i,j}^{\prime }\;-\mathrm{AADP}T_{i}}{\mathrm{AADP}T_{i}}\right|$$

(5) $$\mathrm{MAPE}=\frac{1}{n}\sum_{i}\sum_{j}\mathrm{AP}E_{i,j}$$

where $EF_{i,m,d}$ . is the expansion factor of site i for month ( m ) and day of the week ( d ) obtained from a given method (e.g., from STC site method, single factor method, etc.), $V_{i,j,m,d}^{x}$ is the pedestrian volume for the *j*th STC of site i , which is associated with month ( m ) and day of the week ( d ) for duration x ( x = 8 h in this case), ${\mathrm{AADPT}}_{i,j}^{\prime }$ . is the estimated AADPT at site i for the *j*th STC, $\mathrm{AADP}T_{i}$ is the AADPT of intersection i (calculated using Equation 1), $\mathrm{AP}E_{i,\;j}$ is the absolute percent error at site i for the *j*th STC (note that the total number of j may slightly differ across sites), MAPE . is the MAPE, and *n* is the number of STCs across all sites.

Results are presented based on 8-h STCs obtained from all available days throughout the year (i.e., days when the CC station was functioning properly and they were not excluded by the filtering criteria) and from a subset of days referred to as "candidate" days. Jurisdictions often collect STCs, particularly for signal timing purposes, on days that are typical with respect to vehicular traffic volume. In this work, candidate days refer specifically to non-holiday Tuesdays, Wednesdays, and Thursdays during April, May, June, September, October, and November for the Ontario sites, and from January to May and August to December for Pima County. These months were selected to avoid periods of inclement weather and school holidays.

### Results

It is important to note that the AADPT estimation accuracy when transferring expansion factors using the models to assign factor groups depends on two elements: (a) the quality of factor group classification, shown in Table A3, and (b) the similarity of expansion factors within the same factor group across different jurisdictions. In other words, while classification accuracy can influence the success of transferring expansion factors, the primary factor is the consistency of expansion factors across jurisdictions. This aspect is explored and discussed in more detail in the following sections.

Tables 2 and 3 present the performance of the expansion methods described in the previous section for STCs collected on candidate days and all days, respectively. The results are categorized by overall performance (representing the average of all sites) and by factor group. Minor observations with respect to the tables are provided below, with a discussion on the spatial transferability of expansion factors in the next section.

**Table 2.** Error of Annual Average Daily Pedestrian Traffic (AADPT) Estimates (Mean Absolute Percent Error [MAPE]): Short-Term Counts (STCs) From Candidate Days Only

						Factor group identification methods for each base jurisdiction												
			Reference methods			Waterloo					Toronto			Milton				PC
Target jurisdiction	Observed factor group	#Sites	Ratio 24/8	From STC site	Emp. factor group	SF	MLR	LR	S + C	S	SF	S + C	S	SF	LR	S + C	S	SF
Waterloo (Avg. AADPT = 852)	**Overall**	**101**	**36.8**	**15.5**	**23.6**	**26.0**	**24.7**	**25.4**	**24.9**	**24.5**	**31.9**	**30.7**	**30.5**	**27.5**	**29.3**	**31.7**	**28.8**	**92.3**
	#1: Multipurpose	48	27.8	15.4	23.8	21.9	21.9	23.8	24.4	23.8	24.3	28.6	27.8	23.0	27.8	31.5	27.9	77.1
	#2: Commuter	32	39.4	13.9	22.2	25.3	24.7				33.5			27.3				101
	#3: Schools	21	53.5	18.4	25.5	36.5	31.0	31.4	26.6	28.7	46.8	38.8	40.8	38.0	34.8	32.3	32.3	113
Toronto (Avg. AADPT = 14,282)	**Overall**	**34**	**26.0**	**7.9**	**14.9**	**20.0**	**19.7**	**20.0**	**22.5**	**23.5**	**19.1**	**18.2**	**18.8**	**19.3**	**22.4**	**26.5**	**27.6**	**71.2**
	#1: Multipurpose	21	15.2	7.3	13.3	18.3	17.0	18.8	21.6	23.4	13.2	17.3	17.8	17.8	21.1	26.8	28.2	57.2
	#2: Commuter	7	38.5	8.1	14.0	18.0	23.2				24.4			17.8				92.0
	#3: Schools	6	47.0	9.6	21.2	27.7	24.6	25.3	26.6	23.7	32.4	22.1	23.2	25.8	28.0	24.8	24.8	93.0
Milton (Avg. AADPT = 350)	**Overall**	**33**	**48.8**	**16.9**	**24.3**	**31.8**	**29.2**	**29.7**	**28.1**	**26.9**	**36.3**	**33.0**	**32.6**	**31.2**	**27.8**	**27.7**	**26.1**	**93.3**
	#1: Multipurpose	18	29.9	18.2	24.6	23.7	25.1	24.8	24.1	23.3	23.2	24.0	23.4	23.1	23.1	25.1	24.1	70.1
	#3: Schools	15	71.3	15.3	23.9	41.5	34.2	35.6	32.8	31.3	51.8	43.9	43.6	40.8	33.3	30.7	28.5	121
Pima County (Avg. AADPT = 52)	**Overall**	**20**	**66.8**	**26.1**	**37.2**	**59.4**	**na**	**na**	**na**	**na**	**63.9**	**na**	**na**	**84.8**	**na**	**na**	**na**	**53.9**
	Multipurpose	8	39.0	21.4	27.3	35.5	na	na	na	na	37.0	na	na	55.7	na	na	na	35.4
	S and S	5	119	28.8	48.2	97.3	na	na	na	na	108	na	na	133	na	na	na	106
	Seasonality	7	61.7	29.7	40.8	59.8	na	na	na	na	63.1	na	na	84.1	na	na	na	37.7

*Note*: Emp. = empirical; SF = single factor; MLR = multinomial logistic regression; LR = logistic regression; S + C = school and commercial model; S = school model; PC = Pima County; S and S = school and seasonality; na = not applicable.

Bold cells represent overall results considering all sites for each jurisdiction.

**Table 3.** Error of Annual Average Daily Pedestrian Traffic (AADPT) Estimates (Mean Absolute Percent Error [MAPE]): Short-Term Counts (STCs) From All Days

						Factor group identification methods for each base jurisdiction												
			Reference methods			Waterloo					Toronto			Milton				PC
Target jurisdiction	Observed factor group	#Sites	Ratio 24/8	From STC site	Emp. factor group	SF	MLR	LR	S + C	S	SF	S + C	S	SF	LR	S + C	S	SF
Waterloo (Avg. AADPT = 852)	**Overall**	**101**	**35.5**	**19.1**	**27.9**	**30.9**	**29.0**	**29.7**	**30.4**	**30.7**	**34.2**	**33.3**	**33.6**	**45.5**	**44.8**	**47.0**	**50.3**	**97.6**
	#1: Multipurpose	48	29.5	18.2	26.8	29.9	28.0	28.8	30.0	30.3	30.6	31.9	32.0	45.9	43.8	46.5	50.8	101
	#2: Commuter	32	37.1	18.4	27.0	28.7	27.8				33.5			44.1				102
	#3: Schools	21	46.7	22.1	31.9	36.2	33.3	33.3	31.8	32.3	43.5	38.9	39.6	46.8	48.5	48.8	48.8	84.2
Toronto (Avg. AADPT = 14,282)	**Overall**	**34**	**23.7**	**10.6**	**16.8**	**23.3**	**21.8**	**22.4**	**26.3**	**29.8**	**20.6**	**19.2**	**22.3**	**35.1**	**32.4**	**40.4**	**54.1**	**91.2**
	#1: Multipurpose	21	16.4	9.1	14.2	22.3	19.7	21.5	25.7	30.4	16.9	17.9	21.4	35.9	30.7	40.9	57.7	91.1
	#2: Commuter	7	30.7	11.9	16.9	22.5	23.7				21.5			32.5				100
	#3: Schools	6	39.4	14.4	25.1	27.7	26.6	26.4	28.9	26.9	32.0	25.1	26.2	35.2	39.7	38.2	38.2	81.3
Milton (Avg. AADPT = 350)	**Overall**	**33**	**43.7**	**22.6**	**32.5**	**37.5**	**36.0**	**36.4**	**35.4**	**33.8**	**38.6**	**35.9**	**35.9**	**41.1**	**36.3**	**36.0**	**41.5**	**97.9**
	#1: Multipurpose	18	33.0	21.8	27.2	33.5	33.5	34.2	33.2	31.6	30.5	29.3	29.6	38.6	32.2	32.2	42.7	108
	#3: Schools	15	56.4	23.4	38.8	42.3	39.1	39.1	38.0	36.4	48.1	43.8	43.3	44.0	41.1	40.6	40.1	85.4
Pima County (Avg. AADPT = 52)	**Overall**	**20**	**64.5**	**28.9**	**45.9**	**61.3**	**na**	**na**	**na**	**na**	**64.9**	**na**	**na**	**81.7**	**na**	**na**	**na**	**68.0**
	Multipurpose	8	41.4	22.3	29.9	40.1	na	na	na	na	41.3	na	na	58.4	na	na	na	68.5
	S and S	5	100	35.3	72.6	84.2	na	na	na	na	92.3	na	na	104	na	na	na	83.8
	Seasonality	7	65.5	32.0	45.3	69.3	na	na	na	na	72.3	na	na	92.6	na	na	na	56.1

*Note*: Emp. = empirical; SF = single factor; MLR = multinomial logistic regression; LR = logistic regression; S + C = school and commercial model; S = school model; PC = Pima County; S and S = school and seasonality; na = not applicable.

Bold cells represent overall results considering all sites for each jurisdiction.

The first observation is the noticeable difference in the magnitude of error across different jurisdictions. When considering the most accurate method (from STC site method), the overall MAPE follows the order: Toronto << Waterloo < Milton << Pima County. This pattern correlates with the level of pedestrian activity in each jurisdiction (Table 1): AADPT Toronto >> Waterloo > Milton >> Pima County. The observed order of error magnitude is expected, given that we used a relative measure of error, which tends to yield higher errors at sites with low AADPT (the denominator in Equation 4). If we had used an absolute measure of error, such as the MAE, we would likely observe the opposite pattern. This highlights the importance of the type of error measure employed, as it suggests that direct comparisons across different jurisdictions using these metrics alone may not be entirely fair.

The second observation concerns the methods used as references. When comparing the worst- and best-case scenarios (i.e., ratio 24/8 and from STC site methods, respectively), the ratio 24/8 method, on average, produces an error 2.78 times greater than that of the from STC site method for candidate days. In addition, when expansion factors are grouped into factor groups (empirical factor group method), the overall MAPE for candidate days increases by 8.42%, on average, in absolute terms compared to the from STC site method.

The third observation involves some unexpected results. For example, the school model outperforms the LR model (MAPE = 24.5% versus 25.4%) for candidate days when Waterloo is both the base and target jurisdiction. This is surprising since the LR model provides a better factor group prediction (#1+2 = 93%; #3 = 62%) compared to the school model (#1+2 = 76%; #3 = 67%) (Table A3). A detailed exploration revealed two key factors contributing to this outcome: (a) a limitation in how the expansion factors are calculated. Expansion factors are computed using Equation 2, where for each site, AADPT is divided by the average of the STCs for a given day of the week and month. This formulation does not guarantee minimal averaged errors when expanding STCs to AADPT but instead results in a more even distribution of errors across observations. (b) The expansion factors for factor group #3 consistently exhibit lower values compared to those for the combined factor group #1+2 (Table A3) on candidate days. These lower expansion factors improved the performance of some STCs—particularly those associated with larger volumes, which are likely to exhibit a greater magnitude of error—resulting in lower averaged errors. In other words, the misclassification of sites from factor group #1+2 into #3 accidentally improved the overall expansion performance.

To illustrate this, consider a hypothetical scenario where a site with an AADPT of 300 has two STCs: one with a volume of 100 and another with a volume of 200. The average for the STCs is (100 + 200) / 2 = 150. Thus, an expansion factor of 300 / 150 = 2.00 is obtained. Applying these factors to each STC results in absolute errors of 100 for each STC (e.g., 100 × 2.00 = 200, then 300 – 200 = 100), which translates to percent errors and MAPE of 33.3%. If the expansion factor is arbitrarily reduced to 1.80, we would expect a deterioration in the overall performance, but this does not occur. The STC with a volume of 100 gives an estimated AADPT of 180, resulting in absolute and percent errors of 120 and 40%, respectively. For the STC with a volume of 200, the estimated AADPT is 360 (absolute error = 60, percent error = 20%). The MAPE in this case is 30%, which is lower than the 33.3% obtained using the empirically calculated expansion factor. This illustrates how the formulation used to calculate the expansion factors does not guarantee minimal averaged errors but rather leads to a more even distribution of errors. Note that the same unexpected results would occur if different error metrics—such as the MAE or root mean squared error—were employed.

**Figure 2.**
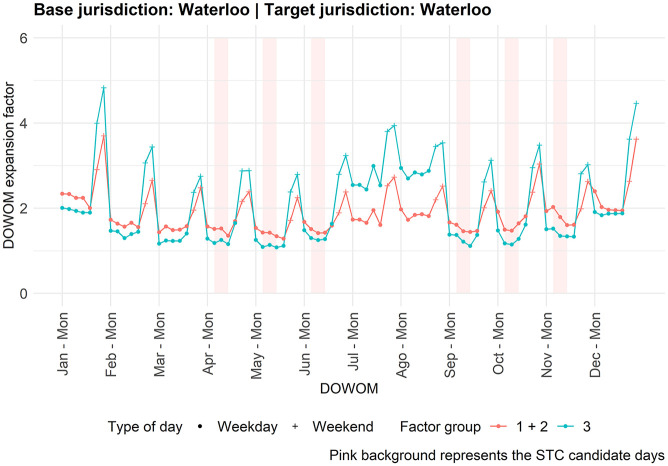
Expansion factors for Waterloo. *Note*: DOWOM = day-of-week-of-month; STC = short-term count.

To further support this finding, when considering all days—a scenario where the differences between expansion factors from the two factor groups are more pronounced (Figure 2), therefore making classification accuracy more important—the LR model outperforms the school model (Table 3: MAPE = 29.7% versus 30.7%).

## Discussion and Conclusions

This section addresses the three practical questions with respect to the spatial transferability of expansion factors posed at the end of the introduction. In addition, it discusses further findings related to cases where the base and target jurisdictions are the same and concludes the work with a summary of key points and practical recommendations. To support the discussion, smaller tables derived from Tables 2 and 3 are used, with a primary focus on STCs collected on candidate days.

Q1: Does the application of expansion factors spatially transferred from another jurisdiction provide sufficiently accurate AADPT estimates to be of practical value?

Short answer: yes, for jurisdictions with similar weather and school holiday periods, transferring expansion factors across jurisdictions provided AADPT estimates of practical value. However, poor results were observed when transferring across jurisdictions with differing characteristics.

Tables 2 and 3 demonstrate that applying expansion factors developed in Pima County to the Ontario jurisdictions resulted in poor performance (MAPE varied from 71.2% to 97.9%). This outcome is attributed to two factors: (a) differences in school holiday months and weather seasonality between the regions, leading to distinct factor group configurations (Figure A1), and (b) the significantly lower pedestrian activity observed in Pima County, which produced expansion factors of different magnitudes compared to those in Ontario. Given the available data, it is challenging to determine the relative influence of each factor. Therefore, it cannot be definitively stated that expansion factors from regions with differing school holiday schedules and weather seasonality are non-transferable.

With respect to the Ontario jurisdictions, Table 4 provides a summary of the spatial transferability using the single factor method. The results are presented for two conditions: average across all sites (“overall”) and just for sites in factor group #3: schools. Column 6 (C6) displays the average MAPE when the base and target jurisdictions differ. For instance, the 29.7% recorded for Waterloo—overall represents the average of 31.9% and 27.5%. C7a compares the values in C6 with the performance when the base and target jurisdictions are the same (i.e., the highlighted cells in C5).

**Table 4.** Summary of Annual Average Daily Pedestrian Traffic (AADPT) Estimation Accuracy Using the Single Factor Method—Candidate Days

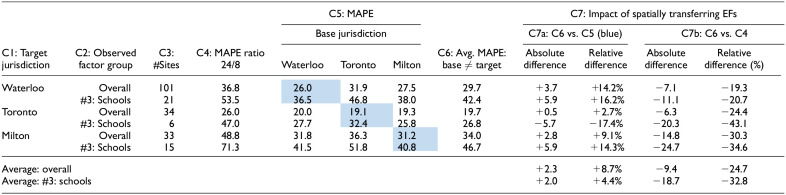

*Note*: Cells highlighted represent the cases where base and target jurisdictions are the same. MAPE = mean absolute percent error; Avg. = average; EF = expansion factor.

The highlighted cells in indicate the base and target jurisdictions are the same.

Overall, the spatial transferability of expansion factors across the Ontario jurisdictions provides good results. When comparing the spatially transferred factors to those obtained when the base and target jurisdictions are the same, there is an average absolute increase in MAPE across the three jurisdictions of just 2.3%, which corresponds to a relative increase of 8.7% (Table 4). If we consider this increase in error as a "penalty" for not having continuous monitoring in the target jurisdiction, it seems to be a relatively minor cost, likely of little practical significance, for estimating AADPT without the need for additional resources to deploy CC stations. This becomes even more apparent when comparing the spatially transferred single factor method with the ratio 24/8 method (C7b), which is probably the method a practitioner would use to cost-effectively convert STCs to daily volumes. On average, the single factor method performs 9.4% better than the ratio 24/8 method in absolute terms.

With respect to factor group #3: schools, a higher magnitude of error is observed both when the base and target jurisdictions are the same and when they are different. Considering the specific characteristics of the expansion factors for sites in this group, it is possible that applying models—and thereby using more accurate expansion factors compared to the single factor method—could improve the performance. This possibility is explored in the next section.

**Figure 3.**
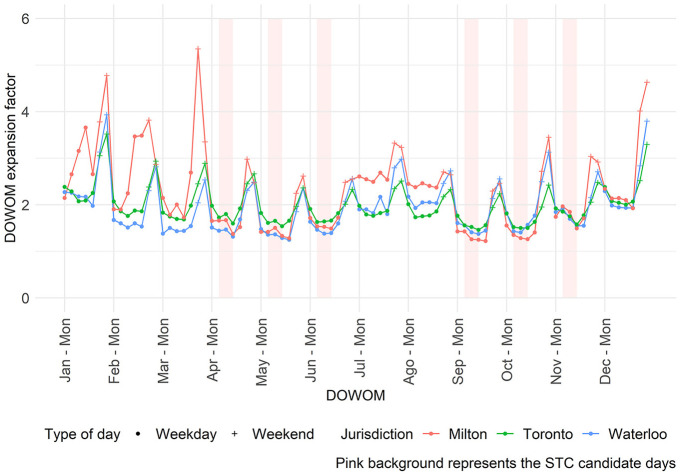
Single factor method expansion factors. *Note*: DOWOM = day-of-week-of-month; STC = short-term count.

To provide better context for Table 4, Figure 3 presents the expansion factors for each Ontario jurisdiction using the single factor method. The expansion factors are notably similar, particularly for candidate days (highlighted in the background), which explains the relatively good performance observed when transferring the factors. In addition, Table 4 shows that the factors from Waterloo and Milton transferred well to other jurisdictions, while those from Toronto produced slightly higher errors. This discrepancy is because Toronto's expansion factors for candidate days are slightly higher than those from Waterloo and Milton (Figure 3), which generates the issue with the MAPE calculation discussed in the previous section. Furthermore, the factors from Milton, when considering all days, differ more and are greater in magnitude than those from Waterloo and Toronto. This difference is reflected in Table 3: for example, the MAPE when Waterloo is the target using the single factor method: (a) base is Waterloo = 30.9%, (b) base is Toronto = 34.2%, and (c) base is Milton = 45.5%.

To consolidate the discussion between all days and candidate days, Table 5 compares the MAPE—in absolute terms—of the single factor method for the two configurations. A greater magnitude of error is anticipated when considering all days instead of candidate days because of the higher variation across the days included in all days. For example, daily pedestrian volumes are expected to vary more across Saturdays in January than across Tuesdays in April. The results for the from STC site method in Tables 2 and 3 confirm this. The spatial transferability between Waterloo and Toronto produced reasonable results when using all days (Table 5), given that the expansion factors for both jurisdictions are relatively similar throughout the year (Figure 3). However, a greater magnitude of error is observed when Milton is the base jurisdiction, and all days are used. The difference in expansion factors depicted in Figure 3 explains this. Overall, this suggests that employing candidate days when spatially transferring expansion factors is recommended.

**Table 5.** Change in Annual Average Daily Pedestrian Traffic Estimation Accuracy (Mean Absolute Percent Error [MAPE]) When Using All Days Versus Candidate Days

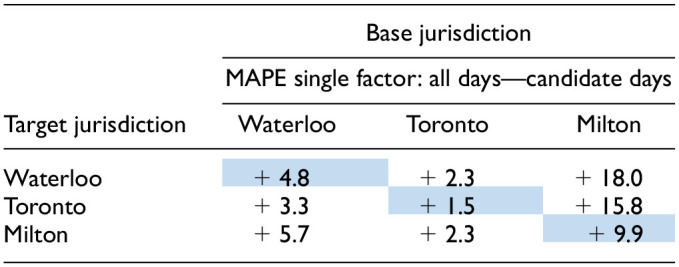

*Note:* Cells highlighted represent the cases where base and target jurisdictions are the same.

To answer the question posed in this section: yes, spatially transferred expansion factors can provide AADPT estimates with sufficient accuracy to be practically useful. This accuracy was achieved when the base and target jurisdictions shared similar weather seasonality and school holidays, and when STCs were conducted on candidate days. However, caution is advised when transferring expansion factors from jurisdictions that meet both conditions but have very low pedestrian activity.

Q2: Does transferring models for associating STC sites with factor groups provide practical benefits over using a single average factor group when transferring expansion factors?

Short answer: the use of models yielded slightly better results than the single average factor group, but this trend was not consistent across all observations. Given the complexity of implementing this approach, the results do not suggest practical benefits.

The results discussed in the previous section highlighted that employing the single factor method may lead to a higher magnitude of error for specific factor groups, such as those associated with school activity. A straightforward solution to this issue, when the base and target jurisdictions are the same, is to use models to assign a factor group to STC sites, thereby applying more accurate expansion factors for each group. However, when the base and target jurisdictions differ, these models must also be transferred from the base jurisdiction, introducing two sources of error: one related to factor group classification and the other to differences in expansion factors within the same factor group across jurisdictions. This section explores whether transferring these models to assign factor groups (modeling method)—and thus applying their associated expansion factors—provides practical benefits over using the single factor method in the context of spatial transferability.

**Figure 4.**
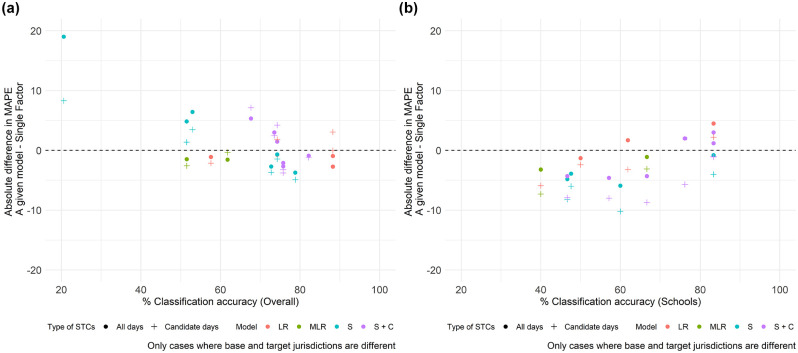
Single factor method versus modeling methods: (*a*) overall and (*b*) factor group #3: schools. *Note*: STC = short-term count; MAPE = mean absolute percent error; MLR = multinomial logistic regression; LR = logistic regression; S + C = school and commercial; S = schools.

Figure 4 summarizes the results from Tables 2 and 3, comparing the performance of the modeling method with the single factor method. The *y*-axis represents the difference in MAPE between the methods: if the value is above zero, the single factor method performs better; if below zero, the modeling method is superior. The *x*-axis indicates the classification accuracy, as extracted from Table A3. Only scenarios where the base and target jurisdictions differ are included.

Figure 4*a* illustrates the overall performance and classification accuracy. It is observed that the differences in performance between all days and candidate days become more pronounced as classification accuracy decreases. This outcome was expected, given that expansion factors for all days differ more across factor groups than those for candidate days. Furthermore, the graph includes 36 observations: 22 show improved performance with the modeling method, while 14 exhibit better results with the single factor method. The 22 "improved" observations show an average absolute improvement in MAPE of 2.02%, whereas the cases where the modeling method generated worse results display an average worsening of 5.14%.

Considering factor group #3: schools (Figure 4*b*), the modeling method showed improved performance in 28 observations, with an average absolute MAPE improvement of 4.53%. In contrast, the single factor method was superior in eight observations, where applying the model led to an average absolute MAPE increase of 2.45%. Compared to the overall results, the modeling method not only outperforms the single factor method more frequently but also with greater impact. This may be of particular interest to practitioners, given the importance of sites located near schools with respect to road safety.

In conclusion, while the modeling method generally tends to produce slightly better results than the single factor method, particularly for sites related to school activities, we do not recommend its use for spatially transferring expansion factors. This recommendation is based on two reasons: (a) the risk that the model could degrade the transferability performance to a greater extent than the potential benefits it might offer, and (b) the added complexity of developing statistical models in the base jurisdiction.

Q3: How does the accuracy of spatially transferring expansion factors compare with that of alternative methods when continuous monitoring is not available?

Short answer: spatially transferred expansion factors performed significantly better than the alternative methods evaluated.

The spatial transferability of expansion factors using the single factor method (using candidate days) is compared to three alternative methods described in the background section: crowd-sourced data (*13*), large language models and satellite imagery (*14*), and the local calibration of DD models (*16*) (Table 6).

**Table 6.** Single Factor Method Compared to Alternative Methods

		Single factor method: target jurisdiction = Waterloo					
			Base jurisdiction		Alternative methods (applied to other jurisdictions)		
Accuracy metric	Volume category	#Sites	Toronto	Milton	Crowd-sourced data (*13*)	Large language models (*14*)	Local calibration of DD models (*16*)
MAPE (%)	Low (<150)	23	41.9	36.8	151	273	NA
	Mid (150–500)	37	31.9	27.1	89	51	NA
	High (500–1000)	14	25.1	21.0	82	51	NA
	Very high (>1000)	27	26.9	23.4	71	49	NA
MAE/ $\bar{Y}$	Overall	101	0.275	0.247	NA	0.50*–*0.75	0.52–0.71

*Note*: DD = direct-demand; MAPE = mean absolute percent error; MAE = mean absolute error; $\bar{Y}$ = average observed AADPT; AADPT = annual average daily pedestrian traffic; NA = not available.

As demonstrated earlier in this work, the level of pedestrian activity at each site and within each jurisdiction directly influences the magnitude of the observed error. To address this and allow direct comparisons across different jurisdictions, the results are presented by volume category. The volume categories used were proposed by StreetLight (*13*). Only the spatial transferability to Waterloo is presented in Table 6 because all sites in Toronto fall into the “very high” category, and some categories in Milton lack a representative sample size. An additional accuracy metric, calculated by dividing the MAE by the average observed AADPT in each jurisdiction (referred to as $\bar{Y}$ ), is also included in Table 6.

The results in Table 6 suggest that the spatial transferability of expansion factors using the single factor method is significantly superior to that of using crowd-sourced data, the ChatGPT approach, and the local calibration of spatially transferred DD models. This highlights the potential of the proposed method, particularly since it relies only on the availability of STCs with pedestrian volumes—commonly obtained through traffic signal design studies—and expansion factors from candidate days in a base jurisdiction with similar school holiday months and weather seasonality.

A few considerations with respect to the results in Table 6: (a) our work used AADPT as the target measure, while the crowd source data and large language model methods used monthly average daily pedestrian traffic (MADPT). The omission of month-to-month seasonality in the MADPT measure might slightly improve the accuracy of these methods compared with if AADPT had been used. (b) In our work, expansion factors and STCs are from the same period. In practical applications, it is common for some sites to only have STCs from previous years, which can introduce a temporal element to the counts and potentially lead to a slightly higher magnitude of error.

### Additional Findings

The additional findings are related to cases where the base and target jurisdictions are the same. Table 7 summarizes the results of the ratio 24/8, empirical factor group, and single factor methods with respect to overall performance and specifically for factor group #3: schools on candidate days. It is observed that grouping all sites into a single factor group increased the MAPE by an average of 4.5% in absolute terms compared to allocating sites to their empirical factor groups. As expected—and consistent with the previous literature (*11*)—this impact was more pronounced for sites in factor group #3 (average absolute increase in MAPE of 13.0%). Furthermore, the single factor method consistently outperformed the ratio 24/8 method. It is important to note that the empirical factor group method is only applicable to sites with continuous monitoring and cannot be applied to STC sites, creating the need to develop models that associate STC sites with appropriate factor groups.

**Table 7.** Summary of Annual Average Daily Pedestrian Traffic Estimation Accuracy When Base and Target Jurisdictions are the Same—Candidate Days

			MAPE			Single factor versus emp. factor group	
Jurisdiction	Configuration	#Sites	Ratio 24/8	Emp. factor group	Single factor (base = target)	Absolute difference	Relative difference (%)
Waterloo	Overall	101	36.8	23.6	26.0	+2.4	+10.1
	#3: Schools	21	53.5	25.5	36.5	+11.0	+42.9
Toronto	Overall	34	26.0	14.9	19.1	+4.2	+28.2
	#3: Schools	6	47.0	21.2	32.4	+11.2	+52.6
Milton	Overall	33	48.8	24.3	31.2	+6.9	+28.5
	#3: Schools	15	71.3	23.9	40.8	+16.9	+70.9
Average: overall						+4.5	+22.2
Average: #3: schools						+13.0	+55.5

*Note*: MAPE = mean absolute percent error; Emp. = empirical.

Table 8 presents the performance of the modeling methods compared to the single factor method. Unlike the spatial transferability cases, where the models did not consistently yield improvements, when the base and target jurisdictions are the same, the modeling methods always show benefits. Modest improvements (0.3–5.1% lower MAPE) are observed in overall performance, while significant improvements (5.5–12.3% lower MAPE) are noted for factor group #3: schools. These results align with the findings from Sobreira and Hellinga (*11*).

**Table 8.** Impact of Modeling Methods on Annual Average Daily Pedestrian Traffic Estimation Accuracy When Base and Target Jurisdictions are the Same—Candidate Days

			MAPE	Absolute difference in MAPE model *i*—single factor			
Base jurisdiction	Configuration	#Sites	Single factor (base = target)	MLR	LR	S + C	S
Waterloo	Overall	101	26.0	−1.3	−0.6	−1.1	−1.5
	#3: Schools	21	36.5	−5.5	−5.1	−9.9	−7.8
Toronto	Overall	34	19.1	na	na	−1.0	−0.3
	#3: Schools	6	32.4	na	na	−10.3	−9.2
Milton	Overall	33	31.2	na	−3.4	−3.5	−5.1
	#3: Schools	15	40.8	na	−7.5	−10.1	−12.3

*Note*: MAPE = mean absolute percent error; MLR = multinomial logistic regression; LR = logistic regression; S + C = school and commercial; S = schools; na = not available.

### Conclusions and Practical Recommendations

Overall, the spatial transferability of expansion factors across jurisdictions with similar school holiday months and weather seasonality has proven effective, making it a promising method for estimating long-term pedestrian volumes in jurisdictions that have STCs but lack sites with continuous monitoring. However, when transferring expansion factors between jurisdictions with differing school holiday schedules and weather patterns, the performance of the spatial transferability was poor. It remains unclear whether this issue arises from differences in factor group configurations or the lower pedestrian activity levels in Pima County that influence the magnitude of the expansion factors. Further research is needed to address this uncertainty.

The spatial transferability of models to assign STC sites to factor groups demonstrated good accuracy of classification. However, the application of expansion factors derived from these models produced inconsistent results: sometimes the modeling methods outperformed the single factor method, and other times the opposite was true. Adding this inconsistency to the complexity of developing the models, we recommend using the single factor method when spatially transferring expansion factors, particularly with STCs collected during periods of typical traffic (i.e., candidate days). Further research considering different jurisdictions is needed to support this recommendation.

The use of spatially transferred expansion factors through the single factor method showed superior performance compared to alternative approaches available to jurisdictions lacking CC sites, such as crowd-sourced data, large language models, or the local calibration of spatially transferred DD models. This further highlights the potential of this method for estimating long-term pedestrian volumes at intersections, as it only requires the availability of STCs—which are relatively easy and inexpensive to collect or may already be available from traffic signal design studies—and the “borrowing” of expansion factors from a similar jurisdiction.

For future research, we recommend the following.

- Assessing the spatial transferability of expansion factors across a wider range of jurisdictions. The "similar" jurisdictions examined in this study are geographically close, potentially sharing habits and customs that may influence pedestrian activity patterns throughout the year. For example, it would be valuable to explore transferability in a jurisdiction within Alberta, Canada, which has similar weather conditions and holiday periods to Ontario. A similar comparison could also be conducted across different U.S. states.
- Further investigating modeling approaches to associate STC sites with factor groups when transferring expansion factors. Examining base jurisdictions with a larger number of CC stations may lead to the development of more robust models, potentially improving their performance and making them a more appealing alternative to the single factor method.
- Assessing the integration of crowd-sourced data to support a more tailored spatial transferability of expansion factors or models to associate STC sites with factor groups.

To conclude this work and encourage further assessment of the spatial transferability of expansion factors in other jurisdictions, Table 9 presents the DOWOM expansion factors on candidate days for the Ontario jurisdictions considered in this work, using the single factor method. These expansion factors should be employed to expand 8-h STCs (7–9 a.m., 11 a.m.–2 p.m., and 3–6 p.m.) on candidate days.

**Table 9.** Day-of-Week-of-Month Expansion Factors: Single Factor Method

		Jurisdiction/expansion factor		
Month	Day of week	Waterloo	Toronto	Milton
April	Tuesday	1.444	1.725	1.662
	Wednesday	1.467	1.800	1.670
	Thursday	1.314	1.597	1.375
May	Tuesday	1.357	1.610	1.416
	Wednesday	1.366	1.653	1.504
	Thursday	1.286	1.540	1.331
June	Tuesday	1.465	1.630	1.537
	Wednesday	1.381	1.642	1.526
	Thursday	1.394	1.660	1.491
September	Tuesday	1.559	1.556	1.428
	Wednesday	1.408	1.524	1.259
	Thursday	1.373	1.461	1.249
October	Tuesday	1.429	1.521	1.352
	Wednesday	1.405	1.502	1.284
	Thursday	1.567	1.502	1.262
November	Tuesday	1.921	1.854	1.966
	Wednesday	1.698	1.751	1.846
	Thursday	1.548	1.576	1.493

## Supplemental Material

sj-docx-1-trr-10.1177_03611981251350639 – Supplemental material for Spatial Transferability of Expansion Factors for Estimating Pedestrian Volume at Intersections Supplemental material, sj-docx-1-trr-10.1177_03611981251350639 for Spatial Transferability of Expansion Factors for Estimating Pedestrian Volume at Intersections by Lucas Tito Pereira Sobreira and Bruce Hellinga in Transportation Research Record
