# Supplementary material for: Spatial Transferability of Expansion Factors for Estimating Pedestrian Volume at Intersections
Source: Transp Res Rec. 2025 Sep 8;2679(11):939–56. doi: 10.1177/03611981251350639 (PMC13046250; doi:10.1177/03611981251350639)
Supplement: sj-docx-1-trr-10.1177_03611981251350639 – Supplemental material for Spatial Transferability of Expansion Factors for Estimating Pedestrian Volume at Intersections [file sj-docx-1-trr-10.1177_03611981251350639.docx]

# APPENDICES

The appendices provide detailed information on the methods and results of Steps 1, 2, and 3.

## A1 – Step 1: Identifying factor groups in the base jurisdiction

### Method

The factor groups in the base jurisdiction are identified using three traffic indicators proposed by Sobreira and Hellinga (11). These indicators, calculated based on the DOWOM expansion factors for each site, aim to distinguish different traffic patterns:

1. Adapted Weekend-Weekday Index (WWI*): this is an adapted version of the WWI proposed by Miranda-Moreno et al. (18). It evaluates the similarity between weekdays and weekends, as detailed in Equations A1 and A2. Equation A2 excludes months affected by school holidays – July and August in Ontario, and June and July in Pima County. These months are omitted because, at sites heavily influenced by school activity, weekday-weekend relationships during school holidays are likely not representative of typical patterns.
2. School Holiday-Typical Index (SHTI): this index identifies sites where pedestrian activity is heavily influenced by school schedules. It measures the pedestrian activity between school holiday periods and typical periods (i.e., months not impacted by school holidays or adverse weather), as described in Equation A3. In Ontario, the typical period encompasses months not affected by winter, from April to November. In Pima County, the typical period excludes months affected by extreme hot weather: August and September. Note that June and July are also affected by hot weather but are the school holiday months. The typical period excludes months with adverse weather, as pedestrian activity may be reduced during these months, making it difficult to isolate the influence of school holidays on typical pedestrian activity.
3. Deviation Across Months (DAM): this indicator measures the standard deviation across months to identify seasonality at each site (Equations A4 and A5). School-holiday months are excluded; therefore, DAM captures only seasonality unrelated to school activity. For example, it can identify sites with reduced pedestrian volume during adverse weather conditions.

WWI*:

${WWI}_{i,m}^{*}= \frac{\frac{1}{2} \sum_{d=6,7} {EF}_{i,m,d}}{\frac{1}{5} \sum_{d=1-5} {EF}_{i,m,d}}$ (A1)

${WWI}_{i}^{*}={median}_{m=1-6,9-12}({WWI}_{i,m})$ (A2)

SHTI:

${SHTI}_{i}= \frac{{median}_{m=7-8;d=1-5}({EF}_{i,m,d})}{{median}_{m=4-6,9-11;d=1-5}({EF}_{i,m,d})}$ (A3)

DAM:

${EF_{Typical}}_{i,m}= {median}_{d=1-5}({EF}_{i,m,d})$ (A4)

${DAM}_{i}={standard deviation}_{m=1-6,9-12}({EF_{Typical}}_{i,m})$ (A5)

Where:

$i$ = a given site

$m$ = month of year

$d$ = day of week (1 = Monday; 7 = Sunday)

${EF}_{i,m,d}$ = DOWOM expansion factor at site $i$

${EF_{Typical}}_{i,m}$ = typical expansion factor at site $i$ and month $m$

Note: the months in the equations represent the calculation for sites in Ontario

The indicators are calculated for each site, and the k-means clustering algorithm is then applied to group sites with similar indicators. The optimal number of clusters (or factor groups) is determined based on two criteria: a) clustering metrics: the total within-cluster sum of squared errors, the silhouette test, and the gap statistic; and b) engineering judgment: the resulting factor groups must make phenomenological sense. In other words, the resulting groups should be – in theory – identifiable through land use, socioeconomic, and transportation attributes, allowing for the development of models in the next step.

Before applying the k-means algorithm, all three indicators were constrained to a maximum value of two to avoid outliers from biasing the clustering procedure due to large indicator values. Additionally, WWI* and SHTI were scaled from 0 to 1. We chose not to scale DAM for two reasons: a) it already ranged from 0 to 1 at almost all sites; and b) it exhibited small variations in some jurisdictions (e.g., from 0.15 to 0.35 in Toronto), so scaling it from 0 to 1 would distort its effect in the clustering process.

### Results

Figure A1 illustrates the factor groups identified in each jurisdiction. Each plot in the figure represents the average (across all sites in the factor group) of the 84 DOWOM expansion factors, with dots arranged chronologically from Monday to Sunday. Vertical gridlines mark the start of each week, aligning with Mondays for each month. The color of the dots differentiates between weekdays and weekends. The y-axis is fixed from 0 to 8 in the Ontario jurisdictions and from 0 to 30 in Pima County.

Three factor groups are observed in Ontario: a) #1 Multipurpose: characterized by relatively consistent pedestrian volume across different months and between weekends and weekdays; b) #2 Commuter: marked by reduced volume on weekends; and c) #3 Primarily School Trips: showing reduced volume on weekends and during school-holiday months. Waterloo and Toronto have all three factor groups, while Milton does not include the #2 Commuter group. Visually, the expansion factors within each factor group appear to have similar magnitudes across the three Ontario jurisdiction, suggesting that these factors may be transferable across the Ontario jurisdictions.

As a comparison, Sobreira and Hellinga (11) found five factor groups when performing a similar analysis in Waterloo. In addition to the three groups observed in the present work, the authors found two additional groups with characteristics similar to #1 Multipurpose and #2 Commuter but with an added seasonality effect: reduced pedestrian volume during the winter months (December-February). This difference may be attributed to the milder winter in 2023-2024 (the period used in this work) compared to the harsher winter in 2022-2023 (the period used by Sobreira and Hellinga (11)).





Figure A1 Resulting factor groups in each jurisdiction

Note: DOWOM = Day-of-week-of-month

As expected, different configurations of factor groups are observed in Pima County. The first factor group, Multipurpose, is similar to the one observed in Ontario. The second factor group also represents a Multipurpose case (i.e., similar volume on weekends and weekdays) but with an added seasonality effect due to the hot summer temperatures. The third factor group combines both school and seasonality effects. In addition to these differences in group configuration, the magnitude of the factors is significantly higher in Pima County than in Ontario. This discrepancy is likely due to the very low pedestrian activity at the sites considered in this work (TABLE 1), rather than a characteristic of general sites in Arizona.

Figure A2 exemplifies the application of the clustering technique in Waterloo. The DAM indicator was also included in the procedure but is not shown in the figure. The clusters are well-defined, with a “dividing threshold” around 1.5 for both WWI* and SHTI.


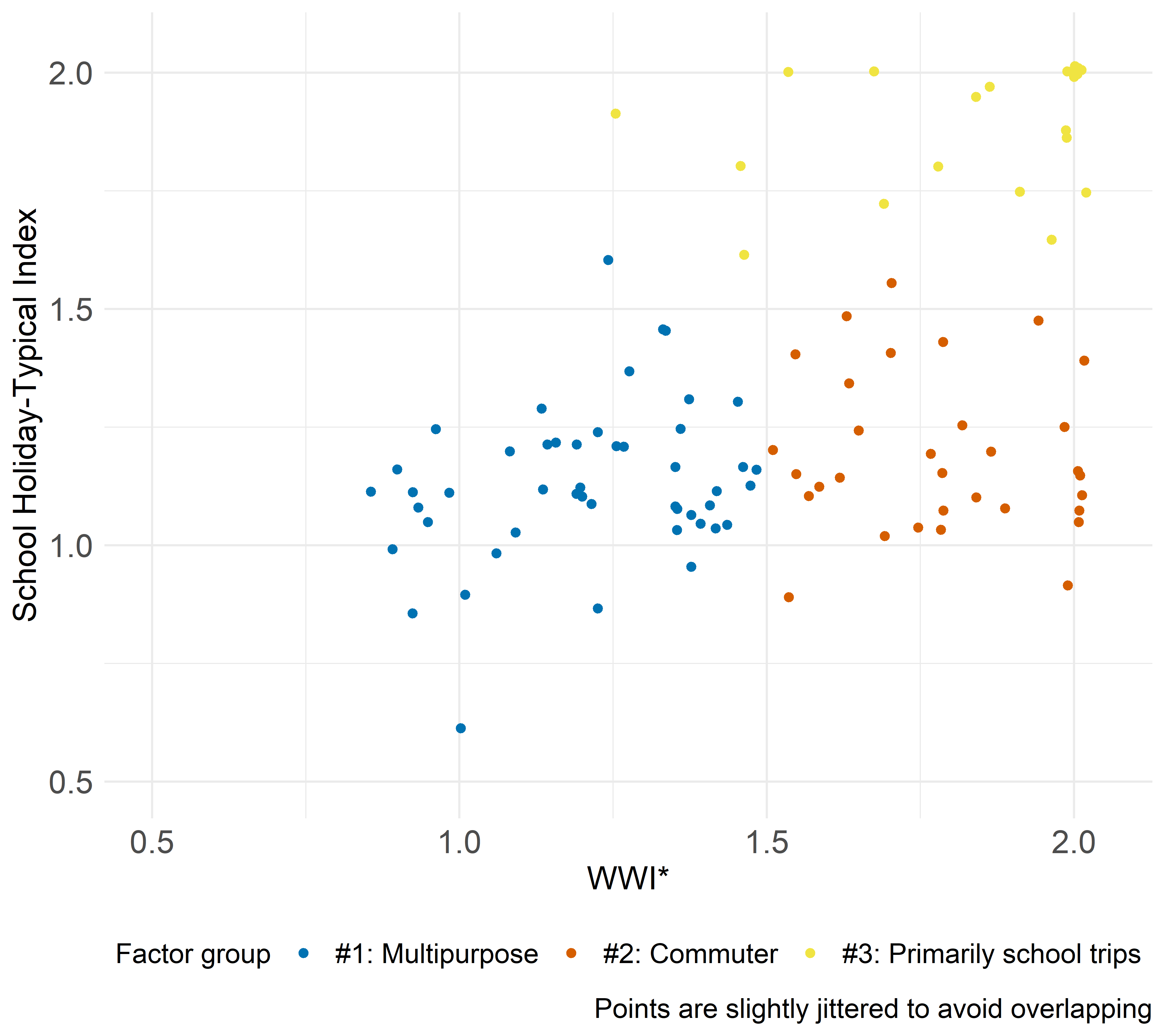


Figure A2 WWI*, SHTI and factor groups in Waterloo

The factor groups assigned to each site in this section are used as the dependent variable in the next section, where models are developed to associate sites with factor groups.

## A2 – Step 2: Modeling factor groups in the base jurisdiction

### Method

In the previous section, the identification of each site’s traffic pattern – and consequently of its factor group – was made possible by the availability of continuous monitoring data at those sites. However, when only STCs are available, accurately identifying the traffic pattern becomes challenging. Therefore, models are developed to associate attributes from the site’s surroundings with a given factor group. It was decided not to develop models for Pima County for two reasons: a) the small sample sizes for each factor group (between 5 and 8 sites) and b) the differing pedestrian activity patterns compared to Ontario sites, as illustrated by the distinct factor group patterns (Figure A1). Given these differences, associating STC sites with factor groups across jurisdictions would not be meaningful.

Regarding the Ontario jurisdictions, Figure A1 shows three factor groups for Waterloo and Toronto, which suggests the development of multinomial logistic regressions (MLRs). However, the sample sizes for factor groups #2 and #3 in Toronto are only 7 and 6 sites, respectively; numbers that are insufficient for proper model calibration. As a result, an MLR was only developed for Waterloo and not for Toronto. In Milton, two factor groups were observed, leading to the use of a binary logistic regression (LR) instead.

The factor groups identified in the previous section were used as the dependent variable in the model. Based on the literature and on the nature of the factor groups, the following explanatory variables were considered: park area, commercial area, employment density, household income, places of worship, residential area, schools (considered in three configurations: all schools, primary schools, and secondary/post-secondary schools), trails, and transit stops. These variables were collected within radii of 100, 200, 400, and 800 meters.

To handle the eleven explanatory variables, which were collected across four different radii, a direct approach would lead to the generation of millions of possible variable sets, and testing each would be impractical. To manage this, a three-step simulation process was employed. First, a radius was randomly assigned to each variable. Second, the linear correlation of every pair of variables was examined, and one variable of the pair was randomly removed if the correlation exceeded 0.50. Third, using the remaining variables, the MLR was calibrated using a stepwise process. This simulation was repeated 100,000 times. The best model (or something close to it), was identified using several criteria: classification accuracy (or confusion matrix), McFadden pseudo-R², and the statistical significance of the variables (p-value < 0.05). Additionally, a 5-fold cross-validation was conducted to assess the model against overfitting and to ensure coefficient stability.

In addition to the MLR and LR, simpler models were used to identify sites in factor group #3: Schools, with other sites categorized as “other factor group”. This approach is motivated by the findings of Sobreira and Hellinga (11), who observed that using the single factor method (i.e., the average of expansion factors from all sites) may be sufficiently accurate for sites in factor groups #1: Multipurpose and #2: Commuter. However, for sites associated with school activities, precise classification into the correct factor group – and thus the use of more accurate expansion factors – plays a crucial role in minimizing AADPT estimation errors. Three simpler models were considered:

1. Binary LR for Waterloo.
2. Presence of schools: a site is categorized into factor group #3 if there is a school within a specified radius. We considered both a) the presence of any school, and b) the presence of only secondary/post-secondary schools. Radii of 100, 200, 400, and 800 meters were tested to determine the optimal classification. In this context, optimal classification refers to the balance between the correct classification of sites in the factor groups “#Others” and “#3: Schools”.
3. Presence of schools and commercial land use: a site is categorized into factor group “#3: Schools” if a school (or secondary/post-secondary school) is within a specified radius and the percentage of commercial land use within the same radius does not exceed a specified threshold. This constraint aims to reduce misclassification where pedestrian activity is influenced not only by a nearby school but also by other land uses. We tested radii of 100, 200, 400, and 800 meters, and commercial land use percentages from 0% to 100% in 5% increments to determine optimal classification.

### Results

TABLE A1 presents a summary of the MLR and LR models. The coefficients are calculated relative to a reference group, indicated in the table. Overall, all coefficients align with theoretical expectations and are consistent with the literature. The MLR achieved a general accuracy of classification of 74.3%, which is slightly higher than the performance reported in previous studies: 67% by Medury et al. (9) and 69% by Sobreira and Hellinga (11). The combination of factor groups #1 and #2 into a single group in the LR improved the general classification, as anticipated for having a simpler model. However, the LR model in Waterloo shows poorer accuracy (61.9%) in classifying sites from factor group #3. The 5-fold cross-validation procedure, repeated 100 times, indicated no signs of overfitting. Finally, all variables had a variance inflation factor (VIF) lower than two, suggesting the absence of multicollinearity.

TABLE A1 Summary of MLR and LR models

| **Variable** |  | **MLR** | | |  | **LR** | | | | |
| --- | --- | --- | --- | --- | --- | --- | --- | --- | --- | --- |
|  |  | **Waterloo  (ref.: #1)** | | |  | **Waterloo  (ref.: #1 + #2)** | |  | **Milton  (ref.: #1)** | |
|  |  | **Radius (m)** | **#2** | **#3** |  | **Radius (m)** | **#3** |  | **Radius (m)** | **#3** |
|  |  |  | **Coef.**  **(SD)** | **Coef.**  **(SD)** |  |  | **Coef.**  **(SD)** |  |  | **Coef.**  **(SD)** |
| Intercept |  | - | -0.506  (0.568) | 1.284  (0.645) |  | - | 0.373  (0.466) |  | - | -0.754  (0.850) |
| Commercial area^1^ |  | 400 | -0.022  (0.019) | **-0.214**  **(0.053)** |  | 400 | **-0.176**  **(0.044)** |  |  |  |
| Employment density^2^ |  |  |  |  |  |  |  |  | 400 | **-1.270**  **(0.532)** |
| Park area^3^ |  | 100 | **-1.869**  **(0.826)** | -0.266  (0.190) |  |  |  |  |  |  |
| Schools - Sec/Post-sec^4^ |  | 200 | **3.484**  **(0.940)** | **4.652**  **(1.125)** |  | 200 | **2.233**  **(0.701)** |  | 800 | **2.569**  **(1.000)** |
| Trails^5^ |  | 100 | **0.594**  **(0.225)** | 0.017  (0.322) |  |  |  |  |  |  |
| Accuracy of classification - General (all data) | | | | 74.3% |  |  | 86.1% |  |  | 84.9% |
| Accuracy of classification - Schools (all data) | | | | 81.0% |  |  | 61.9% |  |  | 80.0% |
| Accuracy of classification - General (5-fold train dataset) | | | | 71.1% |  |  | 85.8% |  |  | 84.8% |
| Accuracy of classification - General (5-fold test dataset) | | | | 62.5% |  |  | 84.1% |  |  | 84.0% |
| McFadden pseudo-R2 |  |  |  | 0.36 |  |  | 0.37 |  |  | 0.36 |
| Sample size |  |  |  | 101 |  |  | 101 |  |  | 33 |
| Ref. = Reference; Coef. = Coefficient. SD = Standard deviation. Statistical significance: bold for p-value < 0.05.  Factor Groups: #1= Multipurpose; #2 = Commuter; #3 = Primarily school trips | | | | | | | | | | |
| ^1^Percentage variable: area of parcels that are commercial / total buffer area × 100. ^2^Employment density per km^2^ / 1,000. ^3^Park area in m^2^ / 1,000. ^4^Count of secondary and postsecondary schools. ^5^Length of multi use trails and paths in km × 10. Adjustments were made to variables to avoid very small or large coefficients | | | | | | | | | | |

TABLE A2 summarizes the school-based models. The performance of the School & Commercial model is comparable to that of the LR model and significantly better than the School model. This highlights the importance of incorporating the commercial component into the school model. For the Waterloo results, the School & Commercial model should be interpreted as follows: a site is classified into factor group “#3: Schools” if a) there is any type of school within 400 meters and b) the commercial area within the 400-meter buffer represents less than 20% of the total buffer area.

TABLE A2 Summary of school-based models

| **Variable** |  | **School & Commercial (Radius / % commercial)** | | | | |  | **School**  **(Radius)** | | | | |
| --- | --- | --- | --- | --- | --- | --- | --- | --- | --- | --- | --- | --- |
|  |  | **Waterloo** |  | **Toronto** |  | **Milton** |  | **Waterloo** |  | **Toronto** |  | **Milton** |
| Commercial area^1^ |  | 400m |  | 200m |  | 400m |  |  |  |  |  |  |
| Commercial area % threshold |  | 20% |  | 25% |  | 25% |  |  |  |  |  |  |
| Schools - All types |  | 400m |  |  |  |  |  | 200m |  |  |  |  |
| Schools - Secondary/Post-secondary |  |  |  | 400m |  | 800m |  |  |  | 200m |  | 800m |
| Accuracy of classification - General |  | 80.2% |  | 94.1% |  | 81.8% |  | 74.3% |  | 73.5% |  | 66.7% |
| Accuracy of classification - Schools |  | 81.0% |  | 83.3% |  | 80% |  | 66.7% |  | 66.7% |  | 86.7% |
| Sample size |  | 101 |  | 34 |  | 33 |  | 101 |  | 34 |  | 33 |
| ^1^Percentage variable: area of parcels that are commercial / total buffer area × 100 | | | | | | | | | | | | |

## A3 – Step 3: Assigning STC sites with a given factor group in the target jurisdiction

Step 3 switches to the target jurisdiction, where only STC sites are available. The models developed in Step 2, based on the base jurisdiction, are transferred and applied to the sites in the target jurisdiction to assign each site to a specific factor group.

TABLE A3 presents the confusion matrices for each model and each base-target pair. To provide a reference, we also included cases where the base and target jurisdictions are the same. On average, the models transferred reasonably well. When the base and target jurisdictions were the same, the average classification accuracy was 78% for factor groups #1, #2, or #1/2, and 76% for factor group #3. When the base and target jurisdictions differed, these accuracies were 70% and 64%, respectively. However, there were specific cases of poor transferability: the MLR model (base: Waterloo) performed poorly when transferred to Toronto (factor group #2) and Milton (factor group #3), and the School model (base: Milton) struggled when transferred to Toronto (factor group #1/2).

TABLE A3 Confusion matrices: transferring models to assign factor groups

| **Target jurisdiction** | **Observed factor group** | **#Sites** |  | **Base jurisdiction (predicted factor group)** | | | | | | | | | | | | | | | | | | | | | | | | | | |
| --- | --- | --- | --- | --- | --- | --- | --- | --- | --- | --- | --- | --- | --- | --- | --- | --- | --- | --- | --- | --- | --- | --- | --- | --- | --- | --- | --- | --- | --- | --- |
|  |  |  |  | **Waterloo** | | | | | | | | | | | |  | **Toronto** | | | | |  | **Milton** | | | | | | | |
|  |  |  |  | **MLR** | | |  | **LR** | |  | **S + C** | |  | **S** | |  | **S + C** | |  | **S** | |  | **LR** | |  | **S + C** | |  | **S** | |
|  |  |  |  | **#1** | **#2** | **#3** |  | **#1/2** | **#3** |  | **#1/2** | **#3** |  | **#1/2** | **#3** |  | **#1/2** | **#3** |  | **#1/2** | **#3** |  | **#1/2** | **#3** |  | **#1/2** | **#3** |  | **#1/2** | **#3** |
| Waterloo | #1: Multipurpose | 48 |  | 40 | 3 | 5 |  | 74 | 6 |  | 64 | 16 |  | 61 | 19 |  | 71 | 9 |  | 65 | 15 |  | 62 | 18 |  | 59 | 21 |  | 36 | 44 |
|  | #2: Commuter | 32 |  | 12 | 18 | 2 |  |  |  |  |  |  |  |  |  |  |  |  |  |  |  |  |  |  |  |  |  |  |  |  |
|  | #3: Schools | 21 |  | 4 | 0 | 17 |  | 8 | 13 |  | 4 | 17 |  | 7 | 14 |  | 9 | 12 |  | 11 | 10 |  | 8 | 13 |  | 5 | 16 |  | 5 | 16 |
|  | Classification accuracy | |  | 83% | 56% | 81% |  | 93% | 62% |  | 80% | 81% |  | 76% | 67% |  | 89% | 57% |  | 81% | 48% |  | 78% | 62% |  | 74% | 76% |  | 45% | 76% |
| Toronto | #1: Multipurpose | 21 |  | 16 | 4 | 1 |  | 27 | 1 |  | 20 | 8 |  | 13 | 15 |  | 27 | 1 |  | 21 | 7 |  | 25 | 3 |  | 18 | 10 |  | 2 | 26 |
|  | #2: Commuter | 7 |  | 6 | 1 | 0 |  |  |  |  |  |  |  |  |  |  |  |  |  |  |  |  |  |  |  |  |  |  |  |  |
|  | #3: Schools | 6 |  | 1 | 1 | 4 |  | 3 | 3 |  | 1 | 5 |  | 1 | 5 |  | 1 | 5 |  | 2 | 4 |  | 1 | 5 |  | 1 | 5 |  | 1 | 5 |
|  | Classification accuracy | |  | 76% | 14% | 67% |  | 96% | 50% |  | 71% | 83% |  | 46% | 83% |  | 96% | 83% |  | 75% | 67% |  | 89% | 83% |  | 64% | 83% |  | 7% | 83% |
| Milton | #1: Multipurpose | 18 |  | 11 | 6 | 1 |  | 13 | 5 |  | 15 | 3 |  | 17 | 1 |  | 18 | 0 |  | 17 | 1 |  | 16 | 2 |  | 15 | 3 |  | 9 | 9 |
|  | #3: Schools | 15 |  | 2 | 7 | 6 |  | 9 | 6 |  | 5 | 10 |  | 6 | 9 |  | 8 | 7 |  | 8 | 7 |  | 3 | 12 |  | 3 | 12 |  | 2 | 13 |
|  | Classification accuracy | |  | 61% | - | 40% |  | 72% | 40% |  | 83% | 67% |  | 94% | 60% |  | 100% | 47% |  | 94% | 47% |  | 89% | 80% |  | 83% | 80% |  | 50% | 87% |

Note: MLR = Multinomial logistic regression; LR = Logistic regression; S + C = School & Commercial; S = School
